# Sex differences in negative affect and postoperative pain in patients undergoing total knee arthroplasty

**DOI:** 10.1186/s13293-019-0237-7

**Published:** 2019-05-06

**Authors:** Meghna Nandi, Kristin L. Schreiber, Marc O. Martel, Marise Cornelius, Claudia M. Campbell, Jennifer A. Haythornthwaite, Michael T. Smith, John Wright, Linda S. Aglio, Gary Strichartz, Robert R. Edwards

**Affiliations:** 1 Department of Anesthesiology, Perioperative and Pain Medicine, Brigham and Women’s Hospital, Harvard Medical School, 75 Francis St, Boston, MA 02115 USA; 2 Connors Center for Women’s Health, Brigham and Women’s Hospital, Boston, MA USA; 3 Brown University School of Medicine, Providence, RI United States; 4 Faculties of Dentistry & Medicine, McGill University, Strathcona Anatomy & Dentistry building, 3640 University Street, Montreal, QC H3A 2B2 Canada; 5 Department of Psychiatry and Behavioral Sciences, Johns Hopkins University School of Medicine, 5510 Nathan Shock Drive, Ste 100, Baltimore, MD 21224 USA; 6 Department of Orthopedic Surgery, Brigham and Women’s Hospital, Harvard Medical School, 75 Francis St, Boston, MA 02115 USA

**Keywords:** Postoperative pain, Sex differences, Total knee arthroplasty, Anxiety, Depression

## Abstract

**Background:**

Knee osteoarthritis (OA) is among the most common and disabling persistent pain conditions, with increasing prevalence in the developed world, and affects women to a greater degree than men. In the USA, the growth of knee OA has been paralleled by an increase in rates of total knee arthroplasty (TKA), a surgical treatment option for late-stage knee OA. While TKA outcomes are generally good, postoperative trajectories of pain vary widely, with some patients reporting a complete absence of pain, but with a significant minority reporting worsening pain. Biopsychosocial factors, including anxiety and depression, are known to contribute importantly to the experience of joint pain, with women reporting a higher degree of negative affective symptoms.

**Methods:**

This study investigated sex differences in TKA outcomes in age-matched groups of men and women at two academic medical centers. Pain and physical function were assessed in 100 patients (50 men and 50 women) during the perioperative period (preoperative visit—6 weeks postsurgical). The association of preoperative negative affect (anxiety and depression scores) to postoperative pain and function was evaluated, with specific attention to sex differences in this relationship.

**Results:**

Overall, women reported more baseline pain-related physical dysfunction (although not higher baseline pain scores), as well as higher acute postoperative pain scores during the 2 weeks following TKA than their male counterparts. By 6 weeks postoperatively, sex differences in reported pain were no longer evident. Interestingly, although women reported higher preoperative levels of emotional distress than men, preoperative anxiety and depression scores were better predictors of severe postoperative pain among men than women, throughout the postoperative test period.

**Conclusions:**

This study underlines the importance of considering sex and psychosocial factors, as well as their interaction, in understanding postsurgical pain trajectories.

## Background

Knee osteoarthritis (OA) is one of the leading causes of pain and disability in the USA and globally, with symptoms ranging from decreased range of motion, stiffness, and limitations in physical mobility, to pain and decreased quality of life, costing hundreds of billions of dollars every year in the USA alone [1–5]. While there is no “cure” for OA, total joint replacement is, for many patients, an effective intervention to reduce pain and improve physical functioning. Perhaps not surprisingly, patients with the most severe pain-related symptomatology and functional impairment are the most likely to pursue total knee arthroplasty (TKA) [2, 6].

Recent decades have brought a growing recognition of the tremendous interpatient variability in pain-related outcomes after surgery [7–11]. Following nearly any operative procedure, including seemingly “minor” surgeries (e.g., [12]), some percentage of patients report persistent postoperative pain [9, 13, 14]. TKA outcomes similarly show great variability, with many patients reporting full resolution of knee pain and large increases in physical capacity, while others report continuing, or even worsening, pain. Recent reviews suggest that approximately 15–25% of TKA patients do not achieve satisfactory outcomes following surgery, prompting interest in identifying the factors that are associated with either positive or negative outcomes [2, 15–19].

Many previous studies have identified differences between men and women in the perception, experience, report, and management of pain, including higher rates of pain, enhanced sensitivity to painful stimuli, and a greater functional impact of pain among women compared to men [20–25]. Clinically, women report more severe OA-related pain and dysfunction compared to their male counterparts with equivalent radiographic findings [4, 26–28]; not surprisingly, more TKA patients are women [6, 16, 29]. Previous cross-sectional and retrospective studies evaluating sex differences in pain and function after joint replacement report greater postoperative pain [30] and surgery-related dysfunction [33] in women. High levels of negative affective symptoms such as anxiety and depression have been associated with greater reports of pain after joint replacement surgery [15, 31–33] and, indeed, after an array of other surgical procedures as well [8, 34–36]. Women are more likely to report high levels of distress and pain-related negative affect [20–23, 37, 38], suggesting a possible mechanism that may contribute to sex differences in pain-related outcomes after TKA.

The present study examined pain-related factors in a cohort of 100 patients undergoing TKA, with age-matched groups of 50 women and 50 men. We investigated sex differences in both the severity and correlates of acute pain following TKA, with a focus on potential psychosocial factors that might contribute to any observed sex differences. Specifically, because previous research has found negative affect (anxiety and depression) to be more prominently associated with more severe pain in women [39–45], we hypothesized that the influence of negative affect on pain would be greater in women following TKA.

## Methods

Subjects in this prospective, longitudinal observational study met the American College of Rheumatology criteria for knee OA and were scheduled to undergo unilateral TKA at either Brigham and Women’s Hospital (Boston, Massachusetts) or Johns Hopkins Bayview Medical Center (Baltimore, Maryland). The institutional review boards of both Brigham and Women’s Hospital and Johns Hopkins University approved all study procedures, and written informed consent was obtained from all participants. Inclusion criteria for the parent study included meeting the American College of Rheumatology criteria for knee OA, being scheduled to undergo unilateral total knee arthroplasty at either Brigham and Women’s Hospital (BWH) Boston, Massachusetts, or Johns Hopkins Hospital, Baltimore, Maryland, age of 45 years or greater, and adequate fluency in English. Exclusion criteria included disorders of cognition preventing completion of the study procedures, recent history of a myocardial infarction, documented peripheral neuropathy of at least moderate severity, severe Raynaud’s, and systematic inflammatory autoimmune disorders. An equal number of male (50) and female (50) patients were selected from this prospective multicenter study, with 25 of each sex selected from each of two participating sites, with specific attention to matching ages between the two groups (men and women). Patients completed psychosocial questionnaires in the preoperative period, approximately 2 weeks before surgery. Patients also reported pain severity and physical function at this preoperative time point, as well as at 6 weeks, and 3 and 6 months after surgery. Additionally, pain severity was assessed at 48 h and 2 weeks postoperatively.

### Pain intensity

The Brief Pain Inventory (BPI; [46, 47]) was used to assess patients’ levels of clinical pain intensity. Patients were asked to rate their “average” and “current” level of pain on an 11-point numeric rating scale with the end points 0 (no pain) and 10 (pain as bad as you can imagine). *Physical function* was assessed using the Western Ontario McMaster Universities Scales (WOMAC), a well-validated, 24-item scale used to assess pain and functional outcomes specific to osteoarthritis, including subscales measuring joint pain and physical/occupational functioning [48, 49]. Higher scores on this scale correspond to worse pain and functioning outcomes.

### Negative affect

Negative affect was assessed using the anxiety and depression short forms from the Patient-Reported Outcomes Measurement Information System (PROMIS), which are widely used and extensively validated [50, 51]. Specifically, we utilized the PROMIS Anxiety Short Form Version 1, which includes seven items scored on a 5-point scale (from “never” to “always”); higher scores represent higher levels of anxiety. We also used the PROMIS Depression Short Form Version 1, which includes eight items scored on a 5-point scale (from “never” to “always”); higher scores represent higher levels of depression.

## Statistical analysis

Analyses were conducted using IBM-SPSS v23 (Chicago, IL). A composite index of negative affect (NA) was calculated by averaging patients’ anxiety and depression scores from the PROMIS. Pain intensity was calculated by averaging patients’ reports of “average” and “current” pain on the BPI. A physical function score was calculated by summing patients’ scores from the WOMAC physical function and stiffness subscales.

Sex differences in pain (BPI) and physical function (WOMAC subscales for physical function and stiffness) were examined using non-parametric independent samples Mann-Whitney *U* tests due to the non-normality of BPI and WOMAC data. An independent samples *t* test was used to examine sex differences in negative affect, and a series of Spearman’s rho correlations was computed to examine associations between negative affect and primary study outcomes due to the non-normality of BPI and WOMAC data.

Univariate analyses were conducted to test the potential confounding influence of demographic (i.e., age, ethnicity, marital status, employment) and clinical (i.e., body mass index, preoperative pain intensity) variables on primary study outcomes (i.e., postop pain at 48 h, 2 weeks, 6 weeks). Variables significantly associated with study outcomes were retained as covariates in the analyses described below.

In order to examine whether the association between negative affect and acute postoperative pain outcomes was moderated by sex, moderation analyses were conducted using the PROCESS macro in SPSS. [52] Separate moderation analyses were conducted using BPI and WOMAC scores at 48 h after surgery as outcomes. In these moderation analyses, a two-way interaction term between negative affect and the potential moderator (i.e., sex) was specified in the models along with the main effects of the IV (i.e., negative affect) and moderator (i.e., sex). A significant two-way interaction effect would suggest that the association between negative affect and pain intensity 48 h after surgery is moderated by patient sex. The same moderation analyses were also performed for pain and functional outcomes at 2 and 6 weeks after surgery. For each of the moderation models described above, bootstrap standard errors (SE) and 95% confidence intervals (CI) were reported.

To adjust for potential family-wise (i.e., type 1) error, Bonferroni-corrected *p* values were calculated. Bonferroni corrections were conducted for each of the primary analyses and took into account the number of analyses conducted per outcome domain. For analyses examining sex differences in acute postoperative pain, three analyses were conducted, so the Bonferroni correction for family-wise error set the alpha level for significance at *p* < 0.016 (i.e., 0.05/3).

## Results

### Sex differences at baseline

Group differences in the sample of men and women are listed in Table 1. Importantly, age, BMI, ethnicity, education, and employment did not differ between sex groups. However, higher anxiety and depression were reported among female patients. Female patients were also less likely to be married.

**Table 1:** Sample characteristics

Variables	Overall sample	Men (*n* = 50)	Women (*n* = 50)	*p*
Age	65.66 (8.82)	67.10 (9.36)	64.20 (8.07)	.63
Ethnicity (%, white)	87%	86%	87.8%	.80
Marital status (%, married)	71%	82%	60%	.02
Employment (%, working)	40%	38%	42%	.68
Body mass index (BMI)	30.92 (6.31)	30.87 (6.18)	30.98 (6.53)	.94
Baseline negative affect (PROMIS)	12.54 (4.43)	11.36 (4.18)	13.78 (4.39)	.003

Values in parentheses are standard deviations

### Association of patient characteristics with pain and dysfunction

Association of patient characteristics (men and women) with pain and disability are shown in Table 2. The mean age of the sample was 65.7 years (SD = 8.8). Older patients reported lower levels of pain 2 weeks after surgery (*R* = − 0.22, *p* < 0.05). Additionally, higher preoperative levels of pain were associated with higher pain at 48 h (*R* = 0.35, *p* < 0.01) and 2 weeks (*R* = 0.36, *p* < 0.01) after surgery and are thus included as covariates in sensitivity analyses described below. Negative affect was significantly associated with preoperative pain (*R* = 0.21, *p* < 0.05) as well as with postoperative pain at 48 h (*R* = 0.30, *p* < 0.01), 2 weeks (*R* = 0.38, *p* < 0.01), and 6 weeks (*R* = 0.29, *p* < 0.01) after surgery. Sensitivity analyses conducted using partial Spearman’s rho correlations indicated that all these effects remained significant even after controlling for age (all *p*’s < 0.05) and presurgical levels of pain (all *p*’s < 0.05). None of the other measured variables were significantly associated with pain or disability (Table 2).

**Table 2:**
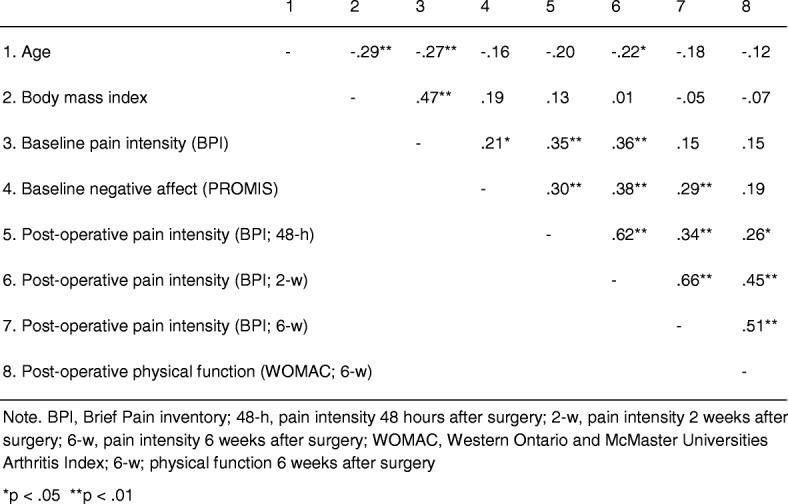
Interrelations among study variables

### Sex differences in postoperative outcomes

**Fig. 1:**
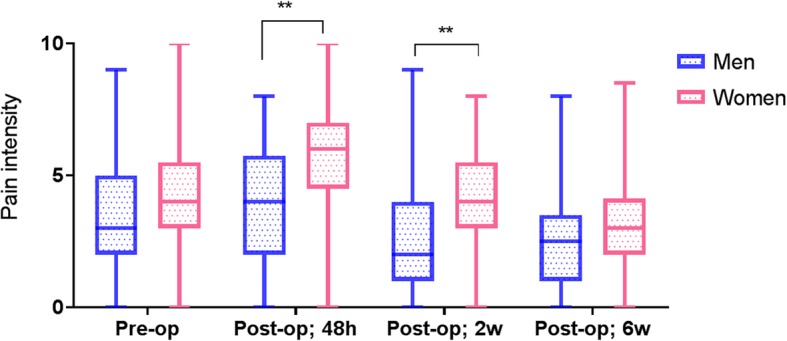
Self-reported pain intensity as a function of time and sex. Pain intensity in male and female patients from presurgery to 6 weeks postsurgery. Data presented as median and interquartile range (IQR)

Sex differences in postoperative pain (BPI) and physical function (WOMAC) were examined using non-parametric independent samples Mann-Whitney *U* tests. Men’s and women’s reported levels of preoperative pain did not differ significantly from each other whereas women reported significantly worse preoperative physical functioning. Women reported significantly greater levels of pain than men at 48 h (*p* < 0.01) and 2 weeks (*p* < 0.01) after surgery (Fig. 1). Sensitivity analyses indicated that these effects remained significant even after controlling for age (both *p*’s < 0.05). Sex differences in pain intensity 48 h after surgery became non-significant after controlling for patients’ presurgical levels of pain (*p* > 0.05), but sex differences in pain observed at 2 weeks remained significant (*p* < 0.05). Pain intensity at 6 weeks, 3 months, and 6 months after surgery was not significantly different between women and men. Men and women also did not report differences in postoperative levels of physical function at 6 weeks, 3 months, and 6 months after surgery.

### Moderating role of sex in the association between negative affect and postoperative pain

**Fig. 2:**
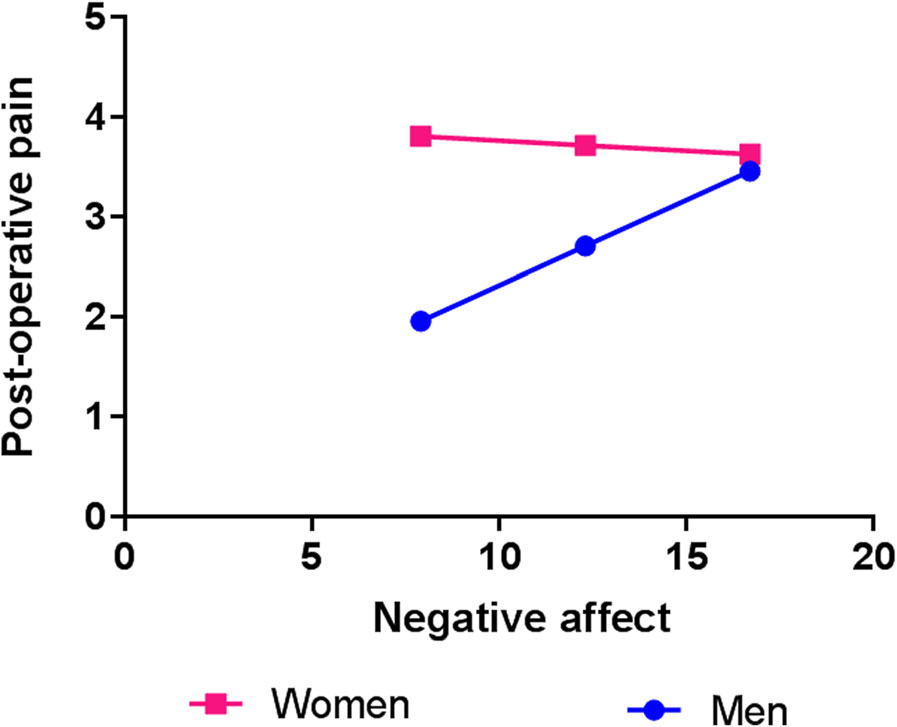
Association between negative affect and pain intensity 2 weeks after surgery. Interaction between sex and baseline negative affect in predicting pain intensity 2 weeks postsurgery

In order to examine sex differences in the relationship between negative affect and acute postoperative pain outcomes, moderation analyses were performed for each of the time points (i.e., 48 h, 2 weeks, 6 weeks) as separate outcome variables. Baseline pain was included as a covariate in the moderation analysis. As can be seen from Table 4, results revealed a significant moderation effect of sex in the association between negative affect and postoperative pain, but only at 2 weeks after surgery. That is, the association between negative affect and 2-week postoperative pain was significantly stronger for men (*R* = 0.50, *p* < 0.01) than women (*R* = 0.05, ns), Fisher’s Z = 2.26, *p* = 0.012 (Fig. 2). Moderation analyses were conducted for 48 h (see Table 3) and 6 weeks (see Table 5) but did not reveal a similar interaction between negative affect and sex at these time points. However, and interestingly, there was a significant sex difference between men’s and women’s interaction of negative affect and pain at all three test times (Tables 3, 4, and 5). Moderation analyses conducted on dysfunction outcomes were not significant (*p* > 0.05).

**Table 3:** Moderating role of sex in the association between negative affect and pain intensity 48 h after surgery

Variables	*β*	Boot SE	*p*	95% CI
*Covariate*				
Baseline pain intensity	0.30	0.12	0.01	LL = 0.07; UL = 0.53
*IVs*				
Negative affect	0.09	0.06	0.12	LL = − 0.02; UL = 0.20
Sex	0.89	0.48	0.07	LL = − 0.07; UL = 1.85
Sex * negative affect	− 0.13	0.11	0.023	LL = − 0.35; UL = 0.09

Values are from the final model

*β* unstandardized regression coefficient, *Boot SE* bootstrapped standard errors, *CI* confidence intervals, *LL* lower limit, *UL* upper limit, *IVs* independent variables

*interaction between

**Table 4:** Moderating role of sex in the association between negative affect and pain intensity 2 weeks after surgery

Variables	*β*	Boot SE	*p*	95% CI
*Covariates*				
Age	− 0.02	0.02	0.29	LL = − 0.07; UL = 0.02
Baseline pain intensity	0.28	0.10	0.01	LL = 0.09; UL = 0.46
*IVs*				
Negative affect	0.08	0.04	0.11	LL = − 0.02; UL = 0.17
Sex	1.01	0.40	0.01	LL = 0.22; UL = 1.80
Sex * negative affect	− 0.19	0.09	0.040	LL = − 0.37; UL = − 0.01

Values are from the final model

*β* unstandardized regression coefficient, *Boot SE* bootstrapped standard errors, *CI* confidence intervals, *LL* lower limit, *UL* upper limit, *IVs* independent variables

*interaction between

**Table 5:** Moderating role of sex in the association between negative affect and pain intensity 6 weeks after surgery

Variables	*β*	Boot SE	*p*	95% CI
*IVs*				
Negative affect	0.12	0.07	0.08	LL = − 0.02; UL =0 25
Sex	1.46	1.25	0.25	LL = − 1.02; UL = 3.94
Sex * negative affect	− 0.08	0.09	0.039	LL = − 0.27; UL = 0.11

Values are from the final model

*β* unstandardized regression coefficient, *Boot SE* bootstrapped standard errors, *CI* confidence intervals, *LL* lower limit, *UL* upper limit, *IVs* independent variables

*interaction between

## Discussion

Given the increasing frequency of TKA, understanding the risk factors for poor pain outcomes after this surgery holds great import. This study investigated the influence of sex on the trajectory of acute pain-related outcomes following TKA, in an age-matched cohort of men and women. Despite similar preoperative pain scores, women reported greater pain at 2 and 14 days after TKA, consistent with previous reports. Women also reported greater preoperative levels of psychological distress (i.e., symptoms of anxiety and depression) than men. Interestingly, however, moderation analysis revealed that preoperative negative affect was a more significant predictor of postoperative pain in men than women (counter to our proposed hypothesis). That is, among this group of TKA patients, we found that women reported higher levels of negative affect, including both anxiety and depression, than men. However, somewhat surprisingly, men exhibited a stronger association between psychosocial distress and pain severity than did women, a sex-related difference that was present throughout the postoperative test period. That is, while a greater negative affect was associated with greater postsurgical pain overall, the pain difference between individuals with low and high negative affect was much greater for men than women (Fig. 2).

Recent years have witnessed substantially increased research regarding sex differences in pain, with multiple reviews concluding that women are at increased risk for an array of acute and persistent pain conditions and that the trajectories of pain treatment outcomes may differ by sex [20–22, 25, 38]. The present findings also suggest that while women and men achieve comparable levels of pain and physical function by 6 weeks postoperatively, women experience greater physical dysfunction preoperatively and more severe pain in the acute postoperative period, consistent with previous studies in joint arthroplasty [60]. Importantly, greater pain in the immediate postoperative period and into the first week following TKA has been associated with an increased likelihood of developing persistent post-TKA pain [19, 36, 53, 54], and this link between severe acute postoperative pain and the development of long-term painful postsurgical complications has also been described for multiple kinds of surgical procedures [9, 13, 55]. In the present study, however, women achieve the same relatively low pain intensity levels as men at 6 weeks following TKA.

Various psychosocial mechanisms may play a fundamental role in shaping sex-related differences in pain. Women tend to report higher levels of general anxiety as well as factors that capture pain-related distress [22, 23, 37, 56]. Several previous reports have suggested that psychosocial variables may contribute to sex differences in pain report, including in the daily intensity of osteoarthritis pain [57, 58]. We found that low levels of negative affect are associated with lower postoperative pain at 2 weeks, but that this is more pronounced in men (Fig. 2). This pattern has been previously observed in both clinic-based studies of chronic pain patients [41, 43] and in longitudinal epidemiologic studies [44, 59]. In both settings, the relationship between pain and mood/anxiety has emerged as stronger among men than women. The mechanisms underlying this sex-related moderation effect are unclear, but the apparent consistency of these findings across settings invites further study. While the presence of sex differences in psychosocial risk factors certainly suggests a plausible pathway by which women might experience more post-TKA pain than men [28, 30] and formed the rationale for our investigations, we in fact found that the association of negative affect and greater pain was driven by men, even if the overall baseline level of such distress is somewhat lower among them. In the present study, we do not have mechanistic data on the physiological or neurobiological mechanisms that might promote a greater impact of negative affect among men, but it is possible that NA-related decrements in pain inhibition are more pronounced among high-NA men undergoing surgery, leaving them more vulnerable to adverse consequences such as increased postoperative pain.

One limitation of the current analysis is that although we used patients’ scores on measures of anxiety and depression to form an index of negative affect, other psychological states (anger, irritability) might also reasonably be included in this index to make it more comprehensive. Another consideration is that the association between negative affect and pain among males was not observed at all time points, and at this point, it is unclear whether this is due to a lack of sufficient power to detect this relationship at those time points, or whether it implies an instability of this relationship.

## Conclusions

As a whole, these findings highlight the complex multidimensional nature of pain, the variability in trajectories of post-TKA pain, and the potential contributory role of sex in shaping these outcomes. Additional work is needed to understand the underlying cause of higher acute postsurgical pain in women. Interestingly, the lack of sex difference in more prolonged postsurgical pain (6 weeks) suggests that despite greater acute pain, potential resilience-related or treatment-related factors may have prevented female patients’ more severe acute pain symptoms from worsening their long-term prognoses. Overall, it is encouraging that women’s pain and function scores improve to the same level as men’s by 6 weeks after surgery.

## Perspectives and significance

The present study found that although women reported both higher preoperative psychosocial distress and acute postoperative pain when compared to men, there was a greater association between preoperative distress and acute postoperative pain in men than women. This relationship requires further investigation but may have implications for the delivery of distress-reducing interventions designed to improve outcomes after joint replacement surgeries, particularly for men with high baseline negative affect. Since severe acute postsurgical pain has consistently been identified as a critical risk factor for longer-term outcomes (i.e., those patients who report the most intense pain in the acute period after surgery are at the greatest risk for chronic pain months or years later) [13, 14, 60, 61], it is important to identify risk/protective variables that shape the experience of pain in the days and weeks following surgery. Collectively, an improvement in the precision of our predictive models will eventually help to develop and deliver targeted, personalized treatments that can reduce the incidence and impact of persistent postoperative pain [9, 13, 62].

## References

[1] Flego A, Dowsey MM, Choong PF, Moodie M (2016). Addressing obesity in the management of knee and hip osteoarthritis - weighing in from an economic perspective. BMC Musculoskelet Disord.

[2] Khan M, Osman K, Green G, Haddad FS (2016). The epidemiology of failure in total knee arthroplasty: avoiding your next revision. Bone Joint J.

[3] Losina E, Paltiel AD, Weinstein AM, Yelin E, Hunter DJ, Chen SP, Klara K, Suter LG, Solomon DH, Burbine SA (2015). Lifetime medical costs of knee osteoarthritis management in the United States: impact of extending indications for total knee arthroplasty. Arthritis Care Res (Hoboken).

[4] Cross M, Smith E, Hoy D, Nolte S, Ackerman I, Fransen M, Bridgett L, Williams S, Guillemin F, Hill CL (2014). The global burden of hip and knee osteoarthritis: estimates from the global burden of disease 2010 study. Ann Rheum Dis.

[5] Gaskin DJ, Richard P (2012). The economic costs of pain in the United States. J Pain.

[6] Wise BL, Niu J, Felson DT, Hietpas J, Sadosky A, Torner J, Lewis CE, Nevitt M (2015). Functional impairment is a risk factor for knee replacement in the multicenter osteoarthritis study. Clin Orthop Relat Res.

[7] Reddi D, Curran N (2014). Chronic pain after surgery: pathophysiology, risk factors and prevention. Postgrad Med J.

[8] Rashiq S, Dick BD (2014). Post-surgical pain syndromes: a review for the non-pain specialist. Can J Anaesth.

[9] Gilron I, Kehlet H (2014). Prevention of chronic pain after surgery: new insights for future research and patient care. Can J Anaesth.

[10] Deumens R, Steyaert A, Forget P, Schubert M, Lavand'homme P, Hermans E, De Kock M (2013). Prevention of chronic postoperative pain: cellular, molecular, and clinical insights for mechanism-based treatment approaches. Prog Neurobiol.

[11] Vandenkerkhof EG, Peters ML, Bruce J (2013). Chronic pain after surgery: time for standardization? A framework to establish core risk factor and outcome domains for epidemiological studies. Clin J Pain.

[12] Slagelse C, Petersen KL, Dahl JB, Finnerup K, Greene K, Leong SP, Levine J, Rowbotham M, Werner MU, Finnerup NB (2014). Persistent postoperative pain and sensory changes following lymph node excision in melanoma patients: a topical review. Melanoma Res.

[13] Schreiber KL, Kehlet H, Belfer I, Edwards RR (2014). Predicting, preventing and managing persistent pain after breast cancer surgery: the importance of psychosocial factors. Pain Manag.

[14] Kehlet H, Jensen TS, Woolf CJ (2006). Persistent postsurgical pain: risk factors and prevention. Lancet.

[15] Lewis GN, Rice DA, McNair PJ, Kluger M (2015). Predictors of persistent pain after total knee arthroplasty: a systematic review and meta-analysis. Br J Anaesth.

[16] Dowsey MM, Gunn J, Choong PF (2014). Selecting those to refer for joint replacement: who will likely benefit and who will not?. Best Pract Res Clin Rheumatol.

[17] Judge A, Arden NK, Cooper C, Kassim JM, Carr AJ, Field RE, Dieppe PA. Predictors of outcomes of total knee replacement surgery. Rheumatology (Oxford). 2012. 10.1093/rheumatology/kes075 22532699

[18] Drosos GI, Triantafilidou T, Ververidis A, Agelopoulou C, Vogiatzaki T, Kazakos K (2015). Persistent post-surgical pain and neuropathic pain after total knee replacement. World J Orthop.

[19] Lavand'homme P, Thienpont E (2015). Pain after total knee arthroplasty: a narrative review focusing on the stratification of patients at risk for persistent pain. Bone Joint J.

[20] Melchior M, Poisbeau P, Gaumond I, Marchand S (2016). Insights into the mechanisms and the emergence of sex-differences in pain. Neuroscience.

[21] Pereira MP, Pogatzki-Zahn E (2015). Gender aspects in postoperative pain. Curr Opin Anaesthesiol.

[22] Bartley E.J., Fillingim R.B. (2013). Sex differences in pain: a brief review of clinical and experimental findings. British Journal of Anaesthesia.

[23] Racine M, Tousignant-Laflamme Y, Kloda LA, Dion D, Dupuis G, Choiniere M (2012). A systematic literature review of 10years of research on sex/gender and pain perception - part 2: do biopsychosocial factors alter pain sensitivity differently in women and men?. Pain.

[24] Racine M, Tousignant-Laflamme Y, Kloda LA, Dion D, Dupuis G, Choiniere M (2012). A systematic literature review of 10years of research on sex/gender and experimental pain perception - part 1: are there really differences between women and men?. Pain.

[25] LeResche L (2011). Defining gender disparities in pain management. Clin Orthop Relat Res.

[26] Glass N, Segal NA, Sluka KA, Torner JC, Nevitt MC, Felson DT, Bradley LA, Neogi T, Lewis CE, Frey-Law LA (2014). Examining sex differences in knee pain: the multicenter osteoarthritis study. Osteoarthr Cartil.

[27] Pope D, El-Othmani MM, Manning BT, Sepula M, Markwell SJ, Saleh KJ (2015). Impact of age, gender and anesthesia modality on post-operative pain in total knee arthroplasty patients. Iowa Orthop J.

[28] Sluka KA, Berkley KJ, O'Connor MI, Nicolella DP, Enoka RM, Boyan BD, Hart DA, Resnick E, Kwoh CK, Tosi LL (2012). Neural and psychosocial contributions to sex differences in knee osteoarthritic pain. Biol Sex Differ.

[29] Katz JN, Bierbaum BE, Losina E (2008). Case mix and outcomes of total knee replacement in orthopaedic specialty hospitals. Med Care.

[30] Mehta SP, Perruccio AV, Palaganas M, Davis AM (2015). Do women have poorer outcomes following total knee replacement?. Osteoarthr Cartil.

[31] Vissers MM, Bussmann JB, Verhaar JA, Busschbach JJ, Bierma-Zeinstra SM, Reijman M (2012). Psychological factors affecting the outcome of total hip and knee arthroplasty: a systematic review. Semin Arthritis Rheum.

[32] Blackburn J, Qureshi A, Amirfeyz R, Bannister G (2012). Does preoperative anxiety and depression predict satisfaction after total knee replacement?. Knee.

[33] Sullivan M, Tanzer M, Stanish W, Fallaha M, Keefe FJ, Simmonds M, Dunbar M (2009). Psychological determinants of problematic outcomes following total knee arthroplasty. Pain.

[34] Sobol-Kwapinska M, Babel P, Plotek W, Stelcer B (2016). Psychological correlates of acute postsurgical pain: a systematic review and meta-analysis. Eur J Pain.

[35] Khan RS, Ahmed K, Blakeway E, Skapinakis P, Nihoyannopoulos L, Macleod K, Sevdalis N, Ashrafian H, Platt M, Darzi A (2011). Catastrophizing: a predictive factor for postoperative pain. Am J Surg.

[36] Masselin-Dubois A, Attal N, Fletcher D, Jayr C, Albi A, Fermanian J, Bouhassira D, Baudic S (2013). Are psychological predictors of chronic postsurgical pain dependent on the surgical model? A comparison of total knee arthroplasty and breast surgery for cancer. J Pain.

[37] Edwards RR, Cahalan C, Mensing G, Smith M, Haythornthwaite JA (2011). Pain, catastrophizing, and depression in the rheumatic diseases. Nat Rev Rheumatol.

[38] Paller CJ, Campbell CM, Edwards RR, Dobs AS (2009). Sex-based differences in pain perception and treatment. Pain Med.

[39] El-Shormilisy N, Strong J, Meredith PJ (2015). Associations between gender, coping patterns and functioning for individuals with chronic pain: a systematic review. Pain Res Manag.

[40] Robinson ME, Dannecker EA, George SZ, Otis J, Atchison JW, Fillingim RB (2005). Sex differences in the associations among psychological factors and pain report: a novel psychophysical study of patients with chronic low back pain. J Pain.

[41] Edwards R, Augustson E, Fillingim R (2003). Differential relationships between anxiety and treatment-associated pain reduction among male and female chronic pain patients. Clin J Pain.

[42] Jones A, Zachariae R, Arendt-Nielsen L (2003). Dispositional anxiety and the experience of pain: gender-specific effects. Eur J Pain.

[43] Edwards RR, Augustson E, Fillingim RB (2000). Sex-specific effects of pain-related anxiety on adjustment to chronic pain. Clin J Pain.

[44] Barry DT, Pilver CE, Hoff RA, Potenza MN (2013). Pain interference and incident mood, anxiety, and substance-use disorders: findings from a representative sample of men and women in the general population. J Psychiatr Res.

[45] Filardo G, Merli G, Roffi A, Marcacci T, Berti Ceroni F, Raboni D, Bortolotti B, Kon E, Marcacci M. Kinesiophobia and depression affect total knee arthroplasty outcome in a multivariate analysis of psychological and physical factors on 200 patients. Knee Surg Sports Traumatol Arthrosc. 2016. 10.1007/s00167-016-4201-3 27329175

[46] Cleeland CS, Ryan KM (1994). Pain assessment: global use of the brief pain inventory. Ann Acad Med Singap.

[47] Tan G, Jensen MP, Thornby JI, Shanti BF (2004). Validation of the brief pain inventory for chronic nonmalignant pain. J Pain.

[48] Beaton DE, Schemitsch E (2003). Measures of health-related quality of life and physical function. Clin Orthop.

[49] Lingard EA, Katz JN, Wright RJ, Wright EA, Sledge CB (2001). Validity and responsiveness of the knee society clinical rating system in comparison with the SF-36 and WOMAC. J Bone Joint Surg Am.

[50] Pilkonis PA, Choi SW, Salsman JM, Butt Z, Moore TL, Lawrence SM, Zill N, Cyranowski JM, Kelly MA, Knox SS (2013). Assessment of self-reported negative affect in the NIH toolbox. Psychiatry Res.

[51] Cella D, Riley W, Stone A, Rothrock N, Reeve B, Yount S, Amtmann D, Bode R, Buysse D, Choi S (2010). The patient-reported outcomes measurement information system (PROMIS) developed and tested its first wave of adult self-reported health outcome item banks: 2005-2008. J Clin Epidemiol.

[52] Hayes AF. Introduction to mediation, moderation, and conditional Process analysis, a regression-based approach methodology in the social sciences. 2nd ed. New York: Guilford Publications; 2017.

[53] Puolakka PA, Rorarius MG, Roviola M, Puolakka TJ, Nordhausen K, Lindgren L (2010). Persistent pain following knee arthroplasty. Eur J Anaesthesiol.

[54] March LM, Cross M, Tribe KL, Lapsley HM, Courtenay BG, Cross MJ, Brooks PM, Cass C, Coolican M, Neil M (2004). Two knees or not two knees? Patient costs and outcomes following bilateral and unilateral total knee joint replacement surgery for OA. Osteoarthr Cartil.

[55] Dahl JB, Kehlet H (2011). Preventive analgesia. Curr Opin Anaesthesiol.

[56] Mogil JS, Bailey AL (2010). Sex and gender differences in pain and analgesia. Prog Brain Res.

[57] Thorn BE, Clements KL, Ward CL, Dixon KE, Kersh BC, Boothby JL, Chaplin WF (2004). Personality factors in the explanation of sex differences in pain catastrophizing and response to experimental pain. Clin J Pain.

[58] Keefe FJ, Lefebvre JC, Egert JR, Affleck G, Sullivan MJ, Caldwell DS (2000). The relationship of gender to pain, pain behavior, and disability in osteoarthritis patients: the role of catastrophizing. Pain.

[59] Regan CO, Kearney PM, Savva GM, Cronin H, Kenny RA (2013). Age and sex differences in prevalence and clinical correlates of depression: first results from the Irish longitudinal study on ageing. Int J Geriatr Psychiatry.

[60] Mauck M, Van d V, Shaw AD (2014). Epigenetics of chronic pain after thoracic surgery. Curr Opin Anaesthesiol.

[61] McNicol ED, Schumann R, Haroutounian S (2014). A systematic review and meta-analysis of ketamine for the prevention of persistent post-surgical pain. Acta Anaesthesiol Scand.

[62] Edwards RR, Dworkin RH, Turk DC, Angst MS, Dionne R, Freeman R, Hansson P, Haroutounian S, Arendt-Nielsen L, Attal N (2016). Patient phenotyping in clinical trials of chronic pain treatments: IMMPACT recommendations. Pain.

